# Valorization of an Old Variety of *Triticum aestivum*: A Study of Its Suitability for Breadmaking Focusing on Sensory and Nutritional Quality

**DOI:** 10.3390/foods12061351

**Published:** 2023-03-22

**Authors:** Alessandro Bianchi, Francesca Venturi, Angela Zinnai, Isabella Taglieri, Basma Najar, Monica Macaluso, Giorgio Merlani, Luciana Gabriella Angelini, Silvia Tavarini, Clarissa Clemente, Chiara Sanmartin

**Affiliations:** 1 Department of Agriculture, Food and Environment, University of Pisa, Via del Borghetto 80, 56124 Pisa, Italy; 2 Interdepartmental Research Centre “Nutraceuticals and Food for Health”, University of Pisa, Via del Borghetto 80, 56124 Pisa, Italy; 3 Pharmacognosy, Bioanalysis and Drug Discovery Unit and Analytical Platform of the Faculty of Pharmacy, Free University of Brussels, Bld Triomphe, Campus Plaine, 1050 Brussels, Belgium

**Keywords:** wheat flour, old variety, sensory quality, VOCs, bread color, nutritional quality, sourdough bread, baker’s yeast bread, dough volume increase

## Abstract

“Avanzi 3-Grano 23” (G23) is an old variety of *Triticum aestivum* from the mountain areas of Lunigiana (north Tuscany, Italy), where traditional farming communities have contributed to its success and on-farm conservation. G23 flour, traditionally used for typical food products, is characterized by particular nutritional and sensory traits but has technological properties which limit its suitability for breadmaking. The aim of this work was to evaluate how to promote the use of G23 through the optimization of bread formulation by leveraging both flour blending and the leavening system. During the preliminary test, three different mixes of G23 flour and a strong flour (C) were tested in terms of their leavening power as a function of leavening agent (baker’s yeast or sourdough). The selected M2 flour, composed of G23:C (1:1 *w*/*w*), was used for further breadmaking trials and 100% C flour was utilized as a control. The sourdough bread obtained with the M2 flour (SB-M2) showed an improved sensory profile compared with the related control (SB-C). Furthermore, SB-M2 exhibited the best aromatic (high content in aldehydes, pyrazines and carboxylic acids) and phytochemical profile (total polyphenols and flavonoids content and antioxidant activity). In contrast, the use of baker’s yeast, although optimal from the point of view of breadmaking, did not result in the same levels of aromatic complexity because it tends to standardize the product without valorizing the sensory and nutritional qualities of the flour. In conclusion, in the experimental conditions adopted, this old wheat variety appears to be suitable for the production of sourdough bakery products.

## 1. Introduction

One of the major cereal crops worldwide is common wheat (*Triticum aestivum*) [1]. Rich in calories, minerals, vitamins, dietary fiber, beneficial bioactive compounds and essential amino acids, wheat and its products are a staple in human nutrition [1,2,3]. However, to meet the industry demands in flour technological quality, modern wheat varieties with higher starch and protein content have been created, and this has led to a consequent decrease in other nutritional components [4,5]. Nevertheless, especially in recent years, consumers are focused on the sensory and nutritional quality aspects of wheat-based products [2,6,7,8]. In this context, old wheat varieties and local landraces have gained increasing attention, and many studies have suggested that they could offer a healthier and a better nutritional profile than modern varieties in terms of protein, lipids, soluble dietary fiber, minerals and different phytochemicals [1,3,9,10,11,12]. However, old wheats are known to have low technological quality, despite their high grain protein percentage, due to their weak gluten index [13,14].

“Avanzi 3-Grano 23” (G23) is an old variety of *Triticum aestivum* cultivated since the early decades of the twentieth century in the mountain areas of Lunigiana (north Tuscany, Italy) [15]. The traditional agricultural communities of this marginal area have contributed for decades to the protection and evolution of this variety of wheat through on-farm conservation to ensure its continuous evolution and diversification to meet the complex agro-environmental conditions and to provide a reliable livelihood and a sustainable food source to local communities [16]. The combined effects of natural and farmer selection have led to a genotype characterized by tall plants, long coleoptiles, early vigor, competition with weeds, cold tolerance, and quality traits suited for local food preferences [15,17].

G23 flour is characterized by particular nutritional and sensory features; nevertheless, it has technological properties which limit its suitability for baking. Indeed, this flour is traditionally used to make local products such as “testaroli” and “panigacci” historically produced in the Lunigiana area [18].

This old variety is now listed as an endangered species as its agricultural production has mostly been interrupted due to the massive focus on the cultivation of modern high-yielding wheat varieties. For these reasons, it is important to create a local food system to promote this variety of wheat through establishing a short supply chain, direct sales, exchange and purchase of specialty agricultural and food products in local markets, which will certainly contribute to a general process of economic revitalization of the territory [19].

A possible approach to increasing G23 economic valorization and thus spreading its use could be the optimization of its flour technological properties by blending with other strong flours and using a suitable leavening agent in order to obtain a bread with nutritional and sensory characteristics particular to this old variety.

Several studies [1,20,21,22] have shown how processes such as sourdough fermentation can boost the phenolic compound availability and antioxidant activity of raw material. In addition, the sourdough induces a high acidity in the final product, prolonging its shelf-life and increasing its nutritional and sensory profile [2,6,22,23].

On the other hand, baker’s yeast is widely used, especially by industrial bakeries, due to its technological properties [24]. Baker’s yeast has the advantage of simplifying the production process, getting the highest yields possible and reducing costs [25].

For these reasons, the aim of this work was to evaluate how to enhance this old wheat variety through the optimization of bread formulation, by leveraging both flour blending and a leavening system.

During the preliminary tests, doughs obtained from different mixes between G23 flour, and a strong flour (C) leavened by brewer’s yeast or sourdough were evaluated considering their volume increase and their fermentation metabolites. At a later stage during further breadmaking trials, the blend of flour selected on the basis of leavening performance was compared with the control (100% strong flour) as a function of the leavening system (brewer’s yeast vs. sourdough). In particular, our attention was focused on the bread’s technological properties, bread sensory profile and compositional traits.

## 2. Materials and Methods

### 2.1. Raw Materials

Control (C) is a wheat flour consisting of a mix of four varieties (Verna, Bologna, Bolero and Pandas) of common wheat (*Triticum aestivum*) supplied by the Department of Agriculture, Food, Environment, and Forestry of the University of Florence.

G23 is a wheat flour of an old variety (“Avanzi 3-Grano 23”) of common wheat (*Triticum aestivum*) and was provided by a small local farmer in Lunigiana (North Tuscany, Italy).

The wheat grains were ground with a commercial mill (Industry-Combi, Waldner Biotech, Lienz, Austria) at the Department of Agriculture, Food, and Environment (DAFE) of the University of Pisa. Table 1 shows the chemical composition and technological features of the flours obtained.

The sourdough used was maintained over one year at the DAFE of the University of Pisa by a daily refreshment procedure as reported by Taglieri et al., 2020 [26], while the baker’s yeast was a commercially available compressed yeast (Zeus Iba s.r.l., Firenze, Italy).

### 2.2. Chemical and Technological Parameters of Flours

The chemical and technological parameters of flours (humidity [27]; ashes [28]; proteins [29]; total fats [30]; falling number [31]; wet gluten and gluten index [32]; dry gluten [33]; total dietary fiber [34]; sugars (maltose; glucose, fructose, sucrose) [35]; total starch [36]; and Chopin alveogram (W, P, L, P/L, G) [37]) were determined through the methods accepted by the International Organization for Standardization (ISO), as previously reported by Bianchi et al., 2022 [6].

### 2.3. Biga Preparation

The leavening and acidifying performances of sourdough were periodically monitored in order to maintain constant and replicable conditions. The bread-making procedure was performed using the “biga” pre-ferment method using sourdough (S) or baker’s yeast (Y).

Sourdough biga (S-biga) was prepared by mixing a strong wheat flour type 0 (56% *w*/*w*), sterile water (33% *w*/*w*), and sourdough (11% *w*/*w*). The mixture was then left to ferment for 18 h at 20 °C. Baker’s yeast biga (Y-biga) was obtained by mixing a strong wheat flour type 0 (68% *w*/*w*), sterile water (31% *w*/*w*), and 1% (*w*/*w*) of baker’s yeast and then fermenting for 21 h at 18 °C. The recipes of S-biga and Y-biga were defined in previous studies [26,38]. The chemical compositions of the two types of biga (S-biga and Y-biga) are reported in Table 2.

### 2.4. Preliminary Leavening Tests

Preliminary leavening tests were conducted using two different types of leavening agent (sourdough (S) and baker’s yeast (Y)) to test different mixes of G23 and C flours (M1 = 1:3 *w*/*w*, M2 = 1:1 *w*/*w*, M3 = 3:1 *w*/*w*) and identify the best blend on the basis of dough volume increase and fermentation metabolites (acetic acid, lactic acid, ethanol). The different doughs (Y-M1, Y-M2, Y-M3, S-M1, S-M2, and S-M3) were prepared following the protocol (formulation, times and temperatures) reported in Section 2.5.

To evaluate the volume increase, as reported in Balestra et al., 2015 [39], 20 g of dough was placed inside a 100 mL graduated cylinder. The dough was left in a prover for 4 h at 32 ± 1 °C. The dough volume increase (DVI) was expressed as a percentage according to the following equation:

(1) $$DVI=\frac{\left(v_{1}-v_{0}\right)}{v_{0}}\times 100$$

where:

*v*_0_ = starting volume of the dough.

*v*_1_ = volume after the leavening time.

### 2.5. Bread Preparation

Two formulations of sourdough bread (SB-M2, SB-C) and two formulation of baker’s yeast bread (YB-M2, YB-C) were produced with water (32%), biga leavening agent (16%), and flour (52%).

The first leavening was allowed to occur for 90 min at 26 ± 1 °C, and then the dough was broken and shaped into 500 g loaves which were left for 2.5 h at 35 ± 1 °C (second leavening). Finally, the loaves were baked at 220 °C for 45 min. The bread was then cooled at room temperature (23 ± 1 °C) and sliced (20 mm) for the analysis.

### 2.6. Physico-Chemical Characterization of Dough, Biga and Bread Samples

The moisture content of samples (dough, biga or bread) was determined on an approximately 5 g sample dried at 105 °C until constant weight. The pH, total titratable acidity (TTA) and the fermentative metabolites (acetic acid, lactic acid, ethanol) were measured as previously reported by Bianchi et al., 2022 [6].

In addition, the flour, biga and bread samples were characterized from a phytochemical point of view. In particular, for total polyphenols, total flavonoids and anti-radical activity evaluation, 80% methanol solution was used to perform a solid/liquid extraction (ratio 1/20 *w*/*v*) from 0.5 g of fresh sample (flour, biga or bread), and the mixture was then sonicated for 30 min. All the extracts were subsequently centrifuged (15 min, 3500 rpm), filtered on a syringe filter (0.45 μm) and stored at 4 °C for immediate analysis.

The Folin–Ciocalteu colorimetric method was applied for the total polyphenols spectrophotometric analysis (wavelength = 765 nm), according to Macaluso et al., 2020 [40], with the results expressed as milligrams of gallic acid equivalents (GAE) per kilogram dry matter (dm) of sample.

The total flavonoids analyses were performed according to the procedure reported by Tavarini et al., 2020 [41], with the results expressed as milligrams of catechin equivalents (CE) per kilogram of sample (dm) and the measures compared with a standard curve of catechin.

The anti-radical activity of the extracts was determined by the DPPH [42], ABTS [43] and FRAP [44] free radical methods. The results were expressed as µmol Trolox equivalents (TE) per gram dm of sample, according to different standard curves of Trolox: in the range 0–200 µmol L^−1^ for the DPPH assay, a range of 0.2–1.5 mM for ABTS and 0–2.0 mM for the FRAP assay.

In order to better evaluate the technological properties of the baked bread, the crumb was analyzed to assess water activity, softness and color, as reported below.

The water activity (a_w_) of the crumb of bread was assessed by a HygroPalm HP23-AW-A device (Rotronic AG, Bassersdorf, Switzerland).

The softness of the crumb of bread was measured as compressibility by a PNR-12 penetrometer (Anton Paar, Rivoli (TO), Italy) using the method described by Taglieri et al., 2021 [38]. Each sample was compressed in five spots by a weight of 90 g for 10 s. The softness was measured in mm of penetration (0.1 mm = 1 penetration unit).

The crumb color of bread was measured according to the CIE L*a*b* color System by means of a tristimulus colorimeter (Eoptis, Mod. CLM-196 Benchtop, Trento, Italy). The Chroma value C* and hue value h* were also calculated as previously reported [45]. The color differences among samples (∆E*_ab_) were calculated as previously reported [38] and expressed in CIELAB units.

The whiteness (WI) and yellowness (YI) indices of the samples were calculated, as reported by Alam et al., 2022 [46], according to the following equations:

(2) $$WI=100-\sqrt{{\left(100-L^{*}\right)}^{2}+{\left(a^{*}\right)}^{2}+{\left(b^{*}\right)}^{2}}$$

(3) $$YI=142.86\times \frac{b^{*}}{L^{*}}$$

### 2.7. Volatile Organic Compounds (VOCs) of Bread

The bread VOC profile (sliced bread samples) was assessed according to the protocol previously described in Sanmartin et al., 2018 [47], sampling the volatile analytes using a 50/30 µm coating thickness SPME (Supelco, St. Louis, MO, USA) and using a gas chromatography-electron impact mass spectrometer (GC-EIMS) (Agilent Technologies Inc., Santa Clara, CA, USA) for their determination.

### 2.8. Sensory Profile of Bread

The bread sensory profile was evaluated by a panel of 8 long-term members of the “Committee of Experts” of DAFE of the University of Pisa, according to the protocol previously described [38], including quantitative (color intensity, presence of lacerations, crumb structure, olfactory intensity, elasticity, resistance to chewing, juiciness, cohesiveness, sapidity, acidity, bitter, aftertaste) and hedonic (visual attractiveness, olfactory pleasantness, tasting pleasantness, global pleasantness) indices. The overall hedonic index of bread was calculated according to Bianchi et al., 2022 [6]. The research obtained the approval of the bioethical committee of the University of Pisa (protocol n. 0088081/2020).

### 2.9. Statistical Analysis

All the evaluations were performed in quadruplicate, and data are reported as mean values ± standard deviation (SD). A one-way ANOVA (CoStat, Cohort 6.0) on the physico-chemical data was performed, followed by the Tukey’s HSD test at *p* ≤ 0.05 significance.

Statistical analysis of volatile organic compounds and hierarchical cluster analysis (HCA) applying the Ward method and using two-way clustering were performed using the JMP Pro 17.0 software package (SAS Institute, Cary, NC, USA). The 3D principal component analysis (PCA) was performed by selecting the three principal components (PCs) obtained by the linear regressions operated on mean-centered, unscaled data.

Sensory analysis data were processed by Big Sensory Soft 2.0 (ver. 2018), carrying out a two-way ANOVA, with samples and panelist as main factors [48], followed by the Friedman test to identify significant descriptors to discriminate samples.

## 3. Results and Discussion

### 3.1. Preliminary Leavening Test

As a first step, we performed a leavening test comparing different percentages of G23 flour and leavening systems to assess their breadmaking suitability through the evaluation of the dough volume increase (DVI) and the fermentative metabolites (Table 3).

During the fermentation time, all the doughs underwent structural changes depending on the fermentation methods used (Y and S). The modifications are related mainly to an increase in resistance to the extension and a decrease in extensibility, with a noticeable change in volume and pH. As shown in Table 3, the two systems differ in terms of DVI, in favor of baker’s yeast, which exhibits a greater leavening power, as reported also in the literature [7,26,39].

The mix M3 (G23:C = 3:1 *w*/*w*) exhibited statistically lower values in terms of DVI, as expected considering the low production of leavening metabolites (acetic acid, lactic acid and ethanol) both for baker’s yeast (Y) and sourdough (S). The other two flour mixes behaved in a similar way when leavened with baker’s yeast (Y-M1 and Y-M2). Conversely, M2 seems to be more favorable for sourdough fermentation than M1 since a higher content of lactic acid was observed in S-M2, with a consequent lower pH, higher TTA, and a significantly higher DVI (Table 3).

According to the results obtained, the ratio 1:1 between G23 flour and C flour allows the use of the greatest amount of G23 with both the leavening systems, resulting in a dough with acceptable results in terms of metabolite content and dough volume.

### 3.2. Physico-Chemical Characterization of Bread

On the basis of the results obtained during the leavening test (see Section 3.1), the M2 flour was thus selected for further breadmaking trials and 100% C flour was utilized as a control. The technological parameters of M2 flour are reported in Table S1. The aim of the breadmaking trial was, therefore, to assess the quality of sourdough bread (SB-M2) and baker’s yeast bread (YB-M2) in comparison with their respective controls (SB-C and YB-C) from compositional, technological and sensory points of view.

As shown in Table 4, the breads obtained with M2 flour (YB-M2 and SB-M2) were characterized by the greatest softness, especially when the sourdough was used.

As expected, the use of sourdough resulted in different TTA and pH levels, due to the production of acetic and lactic acid compared with baker’s yeast [49]. Instead, the M2 seems to be a better substrate for the sourdough since SB-M2 is significantly more acid than SB-C (Table 4). This difference was obviously not observed for the baker’s yeast breads, which did not show any critical issues regardless the flour used.

Regarding the phytochemical features, it is interesting to note that the control breads were generally richer in polyphenols with a consequent higher antioxidant capacity, also derived from its high initial content of C flour (Table 1). However, it is interesting to note that the M2 flour combined with sourdough (SB-M2) underwent a greater increase in phytochemical value from its initial content (Table S1). This increase was not observed for the baker’s yeast breads, which showed lower values than the starting ones. According to [1,3,20], sourdough fermentation promotes the release of bound phenolics, even from flours that showed a lower initial free phenolics content. Clearly, the low pH reached in the sourdough significantly increased the bioavailability of polyphenols in the breads obtained, also enhancing the G23 nutritional and phytochemical potential (Table 4).

### 3.3. Color Characterization of Bread

**Figure 1:**
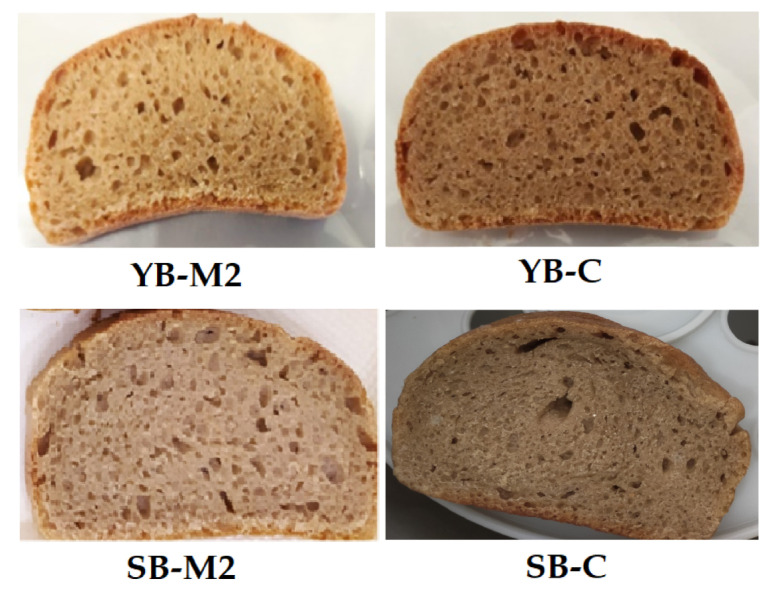
Pictures of the different bread sliced.

Figure 1 includes pictures of the four samples obtained using the two flours (M2 and C) with the two leavening agents (Y and S).

As can be observed in Figure 1, the chromatic characteristics of the samples showed significant differences which seem to be especially related to the flour used, as shown by the comparison of the components a* (≥0 redness; ≤0 greenness) and b* (≥0 yellowness; ≤0 blueness) for both the leavening systems (Table 5).

In general, the lightness (L*) seems to be influenced by the leavening agent and the flour used, as shown by the significant differences in the whiteness index (WI).

The a* index in bakery products is generally related to the Maillard reaction [50], but in our samples, this index is negligible, since in crumb the temperature never exceeds 100 °C and thus the color features of dough are partially retained [51].

The yellow color of crumb depends on both the carotenoid content of the flour and on the baking process that promotes the yellow hue [51].

The yellowness index (YI) of the evaluated flour seems to be more represented in the control samples (YB-C and SB-C), regardless the leavening system.

Moreover, the C samples, having a higher saturation (C*) and a lower hue value (h*), exhibit a color turning to a warm yellow, regardless the leavening system used [13].

Conversely, when M2 flour is used, a significant difference associated with the leavening system is observed, probably due to a higher sensitivity to the pH reduction linked to the use of sourdough [38].

In order to quantify the color differences among the samples, the ΔE*_ab_ values were calculated and are reported in Table 6. All the samples showed a perceptible difference [52], with the greatest color distance (8.89) observed between SB-M2 and YB-C.

A noticeable difference (Table 6) was found even with the same leavening system (SB-M2/SB-C and YB-C/YB-M2), confirming the strong effect of the flour in color determination.

### 3.4. Volatile Organic Profile of Bread Samples

From a purely aromatic point of view, bread is a complex product characterized by a multitude of volatile substances that influence the final aromatic profile. To date, in fact, many studies have focused on evaluating and quantifying the volatile substances of the final aroma of bread, coming to describe over 540 substances produced depending on the type of formulation, type of yeast and cooking method [53,54].

Quantitatively, the analysis of the spontaneous emission of the VOCs allowed the identification of about 62 different compounds (Table S2), where the main groups were alcohols, acids, aldehydes, ketones, esters, pyrazines and pyrrolines, but there were also furans, hydrocarbons and lactones.

**Figure 2:**
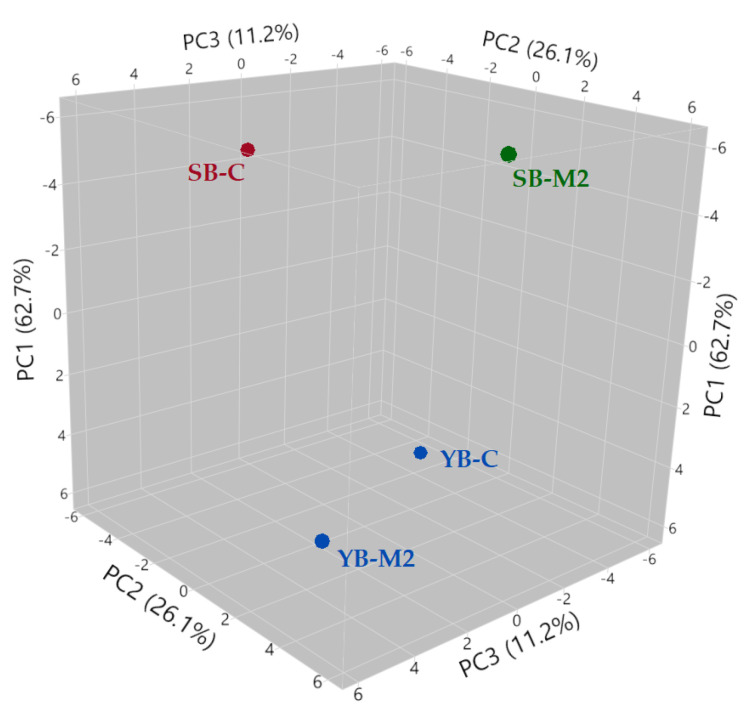
3D version of the principal component analysis (PCA) of volatile organic compounds of the breads.

The analysis of the main components (PC1 + PC2 = total variance of 88.8%) highlights the different volatile substances that allowed differentiation of the loaves into three different groups (Figure 2).

The gas chromatographic analysis shows that the type of leavening agent used led to the production of diverse VOCs. Breads made with baker’s yeast (YB-C and YB-M2) showed a higher production of alcohols and aldehydes with slight differences related to the type of flour: YB-M2 was characterized by a good production of alcohols, such as isobutyl alcohol, 2-methyl-1-butanol, and phenylethyl alcohol, and aldehydes, such as 3-ethyl-2-methyl-1,3-hexadiene, whereas YB-C showed a greater production of aldehydes and alcohols (hexanal and 2-furanomethanol). On the other hand, sourdough breads (SB-C and SB-M2) had more complex VOC profiles with a greater variety of compounds, including pyrazines, pyrimidines, and carboxylic acids. In particular the flour used strongly influenced the volatile expression, allowing clear grouping of the two sourdough breads into separate clusters. (Figure S1). The different flours used have, therefore, led to a different production of volatile substances: (i) SB-M2 was characterized by compounds belonging mainly to aldehydes (i.e., (Z)-2-heptanal, benzaldehyde), pyrazines (i.e., 2,6-dimethylpyrazine and methoxypyrazine), alcohols (i.e., isopentyl alcohol), monoterpenes (i.e., p-cymene); and (ii) SB-C showed mainly alcohols (i.e., 1-pentanol), pyrimidines (i.e., 4-methylpyrimidine) and aldehydes (i.e., 3-methylbutanal).

### 3.5. Sensory Evaluation of Bread

**Figure 3:**
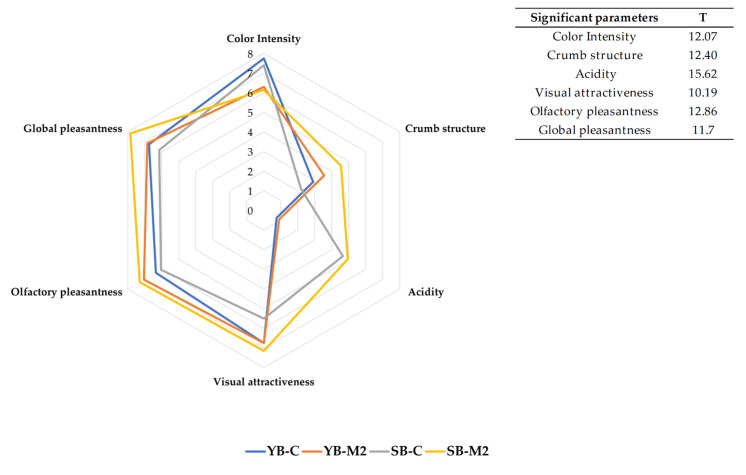
Median of significant qualitative parameters. Friedman’s ANOVA analysis (T > χ^2^; χ^2^ = 9.49).

Figure 3 reports the organoleptic profiles of the breads considering the sensory parameters that showed statistically significant differences.

The sensory profiles are fully in agreement with all the chemical-physical results obtained for all the samples, including color and VOCs, thus confirming that the differences in chemical composition among samples can be clearly perceived by consumers.

In general, the main parameter that enabled differentiation of the breads from a sensory point of view seems to be the formulation. Considering the quantitative parameters, the type of flour clearly influenced the color intensity, but also the crumb structure, while the leavening system strongly affected the acidity. As expected, sourdough breads had a greater perceived acidity, with the highest value reported for the SB-M2 sample, closely followed by SB-C. Regarding the hedonic descriptors, the bread produced with M2 flour was evaluated more positively than that produced with C flour, regardless the leavening agent utilized.

**Figure 4:**
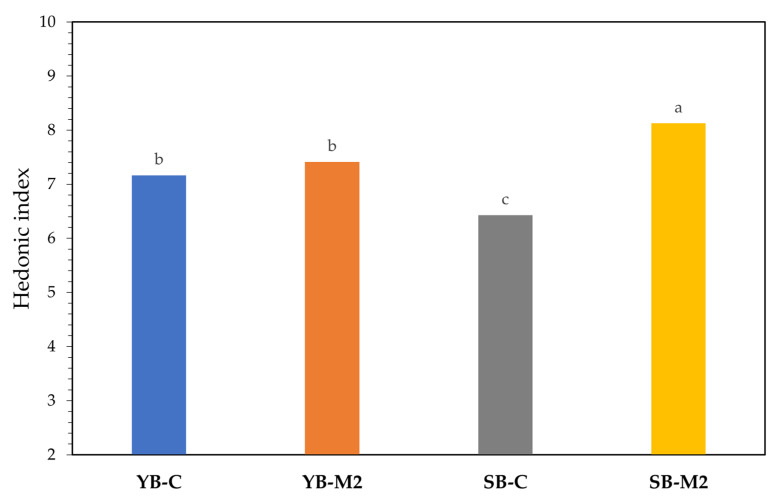
Hedonic index (HI) of different types of bread. Different letters indicate significant differences among samples (*p* < 0.05).

As shown in Figure 4, all the samples received a positive hedonic evaluation (HI > 6). In particular, the highest hedonic index was attributed to the SB-M2 sample, followed by both the breads leavened by baker’s yeast. The baker’s yeast seems to lead to a standardization of the product. For this reason, as also confirmed by the VOCs analysis, the bread showed the same level of aromatic complexity and sensory pleasure.

## 4. Conclusions

The results of this study suggest that the incorporation of flour of an old wheat variety such as “Avanzi 3-Grano 23” along with sourdough fermentation represents an important tool in the development of functional bakery products with improved antioxidant capacity and phenolics bio-accessibility. Moreover, the use of sourdough conferred to the bread a lower pH, due to the higher acidity, which could prolong its shelf-life.

The sourdough bread with the M2 mix in particular, achieved the best results, also from a sensory point of view, reaching a high level of acceptability, as expressed by the hedonic index (HI > 8).

Even if the combination of baker’s yeast and M2 flour was optimal in terms of leavening, it did not achieve the same levels of aromatic complexity and sensory pleasure. According to our results, the baker’s yeast tends to standardize the product, without valorizing the sensory and nutritional potential of the flour

## Figures and Tables

**Table 1:** Chemical and technological parameters of flours (C and G23). Results are expressed as mean ± SD (*n* = 4).

Parameters	Units	C	G23
**Chemical**			
Humidity	% *w*/*w*	10.93 ± 0.30	12.60 ± 0.20
Ashes	% *w*/*w*	1.35 ± 0.09	2.05 ± 0.18
Proteins	% *w*/*w*	12.42 ± 0.32	11.94 ± 0.82
Total fats	% *w*/*w*	2.53 ± 0.63	1.74 ± 0.52
Total dietary fiber	% *w*/*w*	5.72 ± 0.22	3.56 ± 0.32
Maltose	% *w*/*w*	6.28 ± 0.26	3.78 ± 0.36
Glucose	% *w*/*w*	0.43 ± 0.02	0.23 ± 0.05
Fructose	% *w*/*w*	0.14 ± 0.01	0.10 ± 0.03
Sucrose	% *w*/*w*	0.96 ± 0.05	0.46 ± 0.09
Wet gluten	% *w*/*w*	38.82 ± 2.02	29.02 ± 2.23
Dry gluten	% *w*/*w*	12.32 ± 1.62	9.82 ± 1.22
Gluten index	% *w*/*w*	72.22 ± 10.01	41.04 ± 15.12
Total Starch	% *w*/*w*	83.72 ± 0.52	72.54 ± 0.89
Falling number	seconds	333 ± 16	351 ± 18
Total polyphenol	mg GAE/kg dm	800 ± 17	415 ± 12
Total flavonoids	mg CE/kg dm	75.8 ± 0.9	47.4 ± 0.8
ABTS	μmol TE/g dm	1.17 ± 0.07	0.63 ± 0.02
DPPH	μmol TE/g dm	0.70 ± 0.05	0.45 ± 0.03
FRAP	μmol TE/g dm	1.57 ± 0.09	0.44 ± 0.02
**Technological**			
W	10^−4^ joules	263 ± 17	57 ± 14
P	mm	148 ± 14	27 ± 7
L	mm	50 ± 10	88 ± 28
P/L		2.96 ± 0.72	0.36 ± 0.12
G		18.4 ± 1.6	20.8 ± 3.1

**Table 2:** Characterization of the two biga (S-biga and Y-biga) used during the research. Results are expressed as mean ± SD (*n* = 4).

Parameters	Units	S-Biga	Y-Biga
Dry matter	(% dm)	55.20 ± 0.12	59.80 ± 0.16
pH		4.06 ± 0.02	5.43 ± 0.03
TTA	(meq lactic acid/kg dm)	0.117 ± 0.002	0.038 ± 0.003
Acetic acid	(mmol/kg dm)	16.28 ± 0.26	2.56 ± 0.28
Lactic acid	(mmol/kg dm)	91.52 ± 0.62	4.22 ± 0.44
Ethanol	(mmol/kg dm)	56.24 ± 0.24	141.14 ± 0.34

**Table 3:** Physico-chemical composition of six types of dough evaluated.

Parameters	Units	*p*-Value ^1^	Y-M1	Y-M2	Y-M3	S-M1	S-M2	S-M3
Dry matter	(% dm)	*	58.22 ^a^	57.23 ^b^	57.83 ^ab^	53.44 ^d^	54.12 ^c^	53.98 ^cd^
pH		***	5.20 ^b^	5.16 ^b^	5.36 ^a^	3.90 ^e^	3.81 ^d^	4.25 ^c^
TTA	(meq lactic acid/kg dm)	***	0.040 ^d^	0.042 ^d^	0.035 ^e^	0.121 ^b^	0.129 ^a^	0.108 ^c^
Acetic acid	(mmol/kg dm)	**	2.56 ^c^	2.62 ^c^	2.59 ^c^	19.83 ^a^	20.15 ^a^	12.24 ^b^
Lactic acid	(mmol/kg dm)	***	4.71 ^d^	4.62 ^d^	4.42 ^d^	108.52 ^b^	117.54 ^a^	73.5 ^c^
Ethanol	(mmol/kg dm)	**	189.62 ^a^	190.24 ^a^	152.18 ^b^	59.77 ^c^	60.26 ^c^	35.12 ^d^
DVI	%	***	360 ^a^	350 ^a^	180 ^d^	220 ^c^	250 ^b^	70 ^e^

^1^ Significance level: *** *p* < 0.001; ** *p* < 0.01; * *p* < 0.05. In the same row, different letters indicate significant differences among samples.

**Table 4:** Physico-chemical composition of bread.

Parameters	Units	*p*-Value ^1^	YB-M2	YB-C	SB-M2	SB-C
Dry matter	(% dm)	***	56.53 ^a^	55.42 ^b^	53.82 ^d^	54.54 ^c^
Softness	mm	*	2.05 ^ab^	1.81 ^b^	2.15 ^a^	1.73 ^b^
a_w_		ns	0.936	0.938	0.940	0.939
pH		**	5.99 ^a^	6.03 ^a^	4.28 ^d^	4.41 ^c^
TTA	(meq lactic acid/kg dm)	**	0.027 ^c^	0.026 ^c^	0.083 ^a^	0.072 ^b^
Acetic acid	(mmol/kg dm)	**	1.75 ^c^	1.62 ^c^	21.28 ^a^	19.72 ^b^
Lactic acid	(mmol/kg dm)	***	3.96 ^c^	4.27 ^c^	96.59 ^a^	87.4 ^b^
Ethanol	(mmol/kg dm)	**	17.41 ^b^	18.31 ^a^	12.06 ^c^	11.94 ^c^
Total polyphenol	mg GAE/kg dm	***	545 ^d^	618 ^c^	702 ^b^	1054 ^a^
Total flavonoids	mg CE/kg dm	***	72.9 ^d^	84.5 ^c^	109.8 ^b^	113.8 ^a^
ABTS	μmol TE/g dm	***	0.62 ^d^	0.73 ^c^	0.89 ^b^	1.15 ^a^
DDPH	μmol TE/g dm	***	0.36 ^d^	0.42 ^c^	0.55 ^b^	0.72 ^a^
FRAP	μmol TE/g dm	***	0.84 ^d^	0.98 ^c^	1.32 ^b^	1.72 ^a^

^1^ Significance level *** *p* < 0.001; ** *p* < 0.01; * *p* < 0.05; ns: not significant (*p* > 0.05). In the same row, different letters indicate significant differences among samples.

**Table 5:** Chromatic characteristics of bread samples.

Parameters	*p*-Value ^1^	YB-M2	YB-C	SB-M2	SB-C
L*	***	53.69 ^c^	51.05 ^d^	57.32 ^a^	54.85 ^b^
a*	**	3.56 ^b^	5.93 ^a^	3.25 ^b^	5.73 ^a^
b*	***	19.28 ^b^	23.06 ^a^	17.36 ^c^	22.76 ^a^
C*	***	19.60 ^b^	23.81 ^a^	17.66 ^c^	23.47 ^a^
h*	**	79.53 ^a^	75.57 ^b^	79.39 ^a^	75.86 ^b^
WI	***	50.09 ^b^	45.57 ^d^	53.81 ^a^	49.11 ^c^
YI	***	51.30 ^c^	64.53 ^a^	43.27 ^d^	59.28 ^b^

^1^ Significance level *** *p* < 0.001; ** *p* < 0.01. In the same row, different letters indicate significant differences among samples.

**Table 6:** Color differences (ΔE*_ab_) among sliced bread samples. The difference is expressed in CIELAB units. ΔE*_ab_ values up to 2.7 represent chromatic changes perceptible to the human eye.

ΔE*_ab_	YB-C	YB-M2	SB-C	SB-M2
**YB-C**		5.18	3.82	8.89
**YB-M2**			4.26	4.12
**SB-C**				6.44
**SB-M2**				

## Data Availability

Data is contained within the article.

## Supplementary Materials

The following supporting information can be downloaded at: https://www.mdpi.com/article/10.3390/foods12061351/s1, Table S1: chemical and technological parameters of M2 flour. Results are expressed as mean ± SD (*n* = 4); Table S2: complete headspace compositions of baked bread as a function of flour and leavening agent; Figure S1: hierarchical cluster analysis (HCA) based on VOCs of baked bread as a function of flour and leavening agent.
