# Supplementary material for: Valorization of an Old Variety of Triticum aestivum: A Study of Its Suitability for Breadmaking Focusing on Sensory and Nutritional Quality
Source: Foods. 2023 Mar 22;12(6):1351. doi: 10.3390/foods12061351 (PMC10048056; doi:10.3390/foods12061351)
Supplement: Supplementary file 1 [file foods-12-01351-s001.zip › foods-2272180-supplementary.pdf]

Table S1. Chemical and technological parameters of M2 flour. Results are expressed as mean ± SD (n = 4)

Parameters	Units	M2
Chemical		
Humidity	% w/w	11.63 ± 0.22
Ashes	% w/w	1.65 ± 0.12
Proteins	% w/w	12.22 ± 0.26
Total fats	% w/w	2.13 ± 0.43
Total dietary fiber	% w/w	4.72 ± 0.32
Maltose	% w/w	5.28 ± 0.27
Glucose	% w/w	0.33 ± 0.04
Fructose	% w/w	0.16 ± 0.03
Sucrose	% w/w	0.76 ± 0.07
Wet gluten	% w/w	33.92 ± 2.12
Dry gluten	% w/w	11.12 ± 1.42
Gluten index	% w/w	56.73 ± 13.01
Total Starch	% w/w	78.13 ± 0.82
Falling number	Seconds	343 ± 15
Total polyphenol	mg GAE /kg dm	600 ± 27
Total flavonoids	mg CE /g dm	50.7 ± 1.2
ABTS	μmol TE /g dm	0.97 ± 0.07
DPPH	μmol TE /g dm	0.55 ± 0.05
FRAP	μmol TE /g dm	1.09 ± 0.06
Technological		
W	10^{-4} joules	165 ± 17
P	mm	85 ± 14
L	mm	60 ± 10
P/L		1.41 ± 0.72
G		19.6 ± 1.6

Table S2. Complete headspace compositions of baked bread as a function of flour and leavening agent

VOCs compounds	SB-C	SB-M2	YB-C	YB-M2
2,3-epoxybutane	3.70 [a]	0.10 [b]	n.d.	n.d.
acetic acid	19.30 [b]	22.75 [a]	n.d.	n.d.
ethyl acetate	2.70 [a]	n.d.	0.81 [c]	0.95 [b]
2-methyl, 1-propanol	n.d.	n.d.	0.21 [b]	0.90 [a]
isobutyl alcohol	n.d.	n.d.	1.50 [b]	1.75 [a]
3-methyl-butanal	5.10 [b]	6.35 [a]	0.35 [c]	0.12 [d]
2-methyl-butanal	4.55 [a]	1.61 [b]	0.40 [c]	0.22 [d]
pentanal	0.30 [b]	1.45 [a]	0.25 [b]	n.d.
heptane	n.d.	n.d.	n.d.	0.21 [a]
2-ethyl-furan	1.90 [a]	1.15 [b]	0.30 [c]	n.d.
acetoin	n.d.	n.d.	0.25 [b]	0.45 [a]
3-methyl-1-butanol	1.20 [c]	0.40 [d]	17.30 [b]	22.45 [a]
isopentyl alcohol	n.d.	1.40 [a]	n.d.	0.30 [b]
2-methyl-1-butanol	n.d.	n.d.	6.35 [b]	8.05 [a]
1-pentanol	0.95 [a]	0.60 [b]	0.40 [c]	0.30 [d]
1,3,5-cycloheptatriene	0.92 [a]	n.d.	n.d.	n.d.
2,3-butanediol	0.10 [b]	n.d.	0.45 [a]	0.40 [a]
hexanal	14.40 [c]	13.80 [d]	20.90 [a]	18.45 [b]
methyl-pyrazine	n.d.	0.85 [a]	n.d.	0.25 [b]
furfural	3.65 [b]	5.05 [a]	n.d.	0.30 [c]
4-methyl-pyrimidine	n.d.	0.85 [a]	n.d.	n.d.
5-methyl-2-heptanol	n.d.	n.d.	0.25 [a]	0.10 [b]
2-furanmethanol	n.d.	n.d.	0.95 [b]	1.25 [a]
p-xylene	n.d.	n.d.	1.45 [a]	0.70 [b]
m-xyleme	n.d.	n.d.	0.65 [b]	1.00 [a]
1-hexanol	2.05 [d]	3.50 [c]	4.95 [a]	4.40 [b]
2-heptanone	n.d.	n.d.	0.45 [b]	1.30 [a]
4-heptanone	1.20 [a]	0.65 [c]	1.10 [b]	0.70 [c]
2n-butylfuran	0.65 [a]	0.50 [b]	n.d.	n.d.
heptanal	2.95 [a]	2.40 [c]	1.90 [d]	2.75 [b]
2-methoxy pyrazine	1.30 [b]	1.35 [b]	1.85 [a]	1.10 [c]
2,5-dimethyl-pyrazine	1.05 [a]	0.20 [b]	0.10 [c]	0.05 [c]
ethyl-pyrazine	n.d.	0.75 [a]	n.d.	0.45 [b]
2,3-dimethyl-pyrazine	n.d.	n.d.	0.15 [b]	0.20 [b]
2,6-dimethyl pyrazine	0.05 [b]	0.35 [a]	0.10 [b]	0.40 [a]
α-pinene	0.60 [a]	0.40 [c]	0.50 [b]	0.15 [d]
(E)-2-heptenal	0.15 [b]	0.40 [a]	n.d.	n.d.
(Z)-2-heptanal	n.d.	0.75 [a]	0.10 [b]	0.05 [b]
benzaldehyde	0.30 [d]	1.95 [a]	0.40 [c]	0.55 [b]
5-methylfurfural	0.30 [a]	n.d.	n.d.	0.05 [b]
1-octen-1-ol	0.20 [c]	n.d.	0.45 [a]	0.35 [b]
hexadecanoic acid	n.d.	0.30 [a]	n.d.	n.d.
2-pentyl furan	20.50 [b]	15.45 [d]	24.30 [a]	18.25 [c]

δ-3-carene	0.50 [b]	0.75 [a]	n.d.	n.d.
p-cymene	0.35 [b]	0.90 [a]	n.d.	n.d.
limonene	1.10 [a]	1.15 [a]	0.85 [b]	0.90 [b]
3-ethyl-2-methyl-1,3-hexadiene	n.d.	n.d.	1.45 [a]	0.95 [b]
1-acetyl-1,4-dihydropyridine	n.d.	n.d.	0.05 [b]	0.15 [a]
5-ethylcyclopentene-1-carbaldehyde	1.90 [b]	6.10 [a]	n.d.	n.d.
phenylethanol	0.70 [b]	1.25 [a]	n.d.	n.d.
3,5,5-trimityl-1-hexanol	n.d.	n.d.	0.35 [a]	0.25 [a]
(E)-2-octenal	0.55 [b]	0.75 [a]	0.25 [c]	0.25 [c]
nonanal	0.90 [d]	1.65 [c]	2.65 [a]	1.80 [b]
phenylethyl alcohol	n.d.	n.d.	2.10 [b]	4.10 [a]
ethyl caprylate	n.d.	n.d.	0.35 [b]	0.25 [b]
decanal	n.d.	n.d.	0.10 [b]	0.10 [b]
(E,Z),2,4-decadienal	0.35 [a]	n.d.	n.d.	n.d.
(E,E)-2,4-decadienal	1.50 [a]	n.d.	0.90 [b]	0.20 [c]
1-methoxy-4-methylbicyclo(2,2,2)octane	n.d.	0.25 [a]	n.d.	n.d.
dihydro-5-pentyl-2(3H)-furanone	n.d.	n.d.	0.20 [a]	0.15 [a]
trans-9-decalol	0.30 [a]	0.35 [a]	n.d.	n.d.
1H-indene,2,3-dihydro-1,1,3-trimethyl-3-phenyl	n.d.	n.d.	0.11 [b]	0.21 [a]
**Total**	98.22	98.46	98.48	98.21

n.b.= not determinated. In the same row, different letters indicate significant differences among samples.

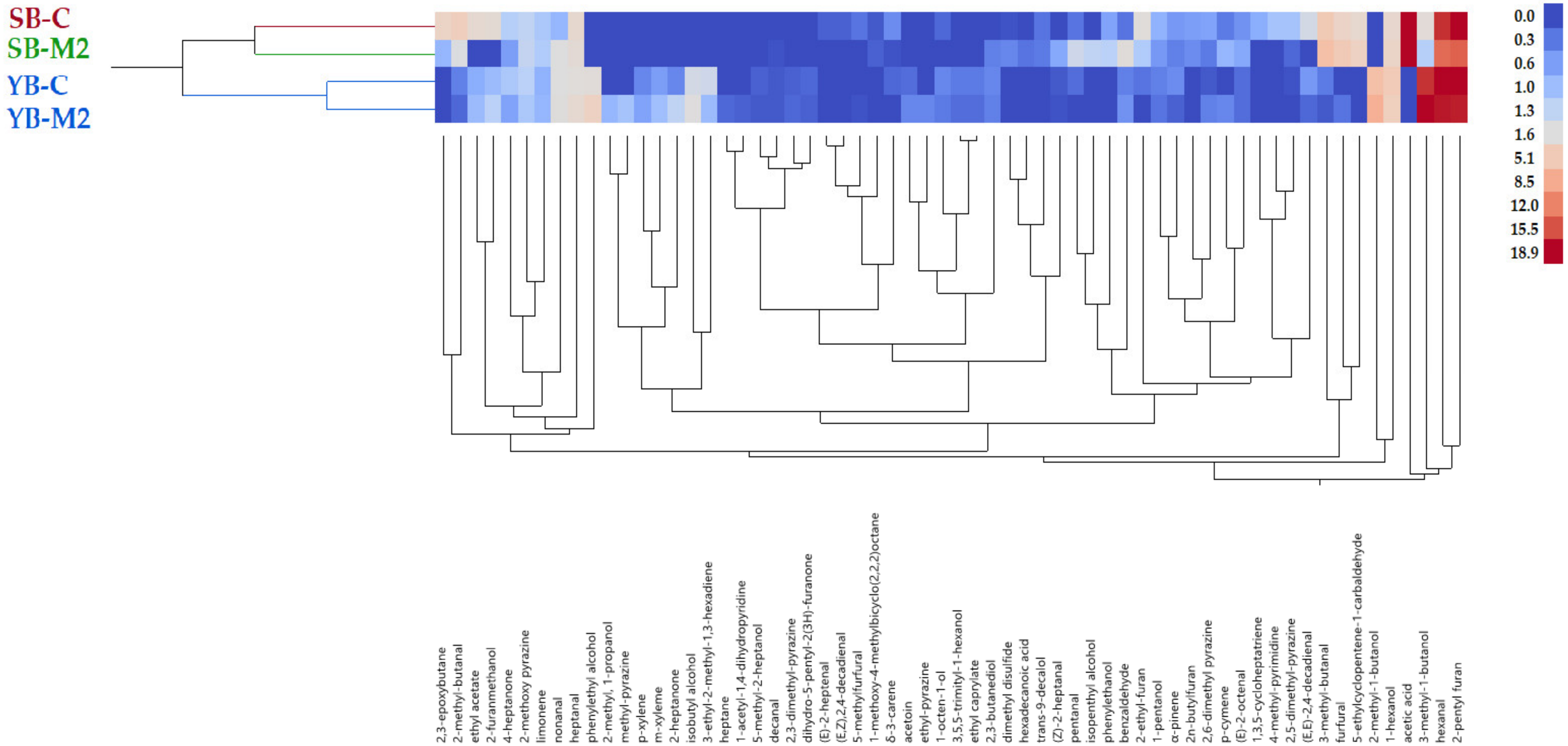

Figure S1. Hierarchical cluster analysis (HCA) based on VOCs of baked bread as a function of flour and leavening agent.
